# First whole-genome sequence and assembly of the Ecuadorian brown-headed spider monkey (*Ateles fusciceps fusciceps*), a critically endangered species, using Oxford Nanopore Technologies

**DOI:** 10.1093/g3journal/jkae014

**Published:** 2024-01-19

**Authors:** Gabriela Pozo, Martina Albuja-Quintana, Lizbeth Larreátegui, Bernardo Gutiérrez, Nathalia Fuentes, Felipe Alfonso-Cortés, Maria de Lourdes Torres

**Affiliations:** Laboratorio de Biotecnología Vegetal, Colegio de Ciencias Biológicas y Ambientales, Universidad San Francisco de Quito (USFQ), Quito 170901, Ecuador; Instituto Nacional de Biodiversidad (INABIO), Quito 170135, Ecuador; Laboratorio de Biotecnología Vegetal, Colegio de Ciencias Biológicas y Ambientales, Universidad San Francisco de Quito (USFQ), Quito 170901, Ecuador; Laboratorio de Biotecnología Vegetal, Colegio de Ciencias Biológicas y Ambientales, Universidad San Francisco de Quito (USFQ), Quito 170901, Ecuador; Laboratorio de Biotecnología Vegetal, Colegio de Ciencias Biológicas y Ambientales, Universidad San Francisco de Quito (USFQ), Quito 170901, Ecuador; Department of Biology, University of Oxford, Oxford OX1 3SZ, UK; Proyecto Washu/Fundación Naturaleza y Arte, Quito 170521, Ecuador; Proyecto Washu/Fundación Naturaleza y Arte, Quito 170521, Ecuador; Laboratorio de Biotecnología Vegetal, Colegio de Ciencias Biológicas y Ambientales, Universidad San Francisco de Quito (USFQ), Quito 170901, Ecuador; Instituto Nacional de Biodiversidad (INABIO), Quito 170135, Ecuador

**Keywords:** whole-genome sequence, genome assembly, reference genome, spider monkey, *Ateles fusciceps fusciceps*, Oxford Nanopore sequencing

## Abstract

The Ecuadorian brown-headed spider monkey (*Ateles fusciceps fusciceps*) is currently considered one of the most endangered primates in the world and is classified as critically endangered [International union for conservation of nature (IUCN)]. It faces multiple threats, the most significant one being habitat loss due to deforestation in western Ecuador. Genomic tools are keys for the management of endangered species, but this requires a reference genome, which until now was unavailable for *A. f. fusciceps*. The present study reports the first whole-genome sequence and assembly of *A. f. fusciceps* generated using Oxford Nanopore long reads. DNA was extracted from a subadult male, and libraries were prepared for sequencing following the Ligation Sequencing Kit SQK-LSK112 workflow. Sequencing was performed using a MinION Mk1C sequencer. The sequencing reads were processed to generate a genome assembly. Two different assemblers were used to obtain draft genomes using raw reads, of which the Flye assembly was found to be superior. The final assembly has a total length of 2.63 Gb and contains 3,861 contigs, with an N50 of 7,560,531 bp. The assembly was analyzed for annotation completeness based on primate ortholog prediction using a high-resolution database, and was found to be 84.3% complete, with a low number of duplicated genes indicating a precise assembly. The annotation of the assembly predicted 31,417 protein-coding genes, comparable with other mammal assemblies. A reference genome for this critically endangered species will allow researchers to gain insight into the genetics of its populations and thus aid conservation and management efforts of this vulnerable species.

## Introduction

The brown-headed spider monkey (*Ateles fusciceps fusciceps*) is a neotropical primate inhabiting northwestern Ecuador (its presence in Colombia is uncertain). It is most commonly found below 1,200 masl, but its altitudinal range can go as high as 2,300 masl (Gallo-Viracocha *et al*. 2022). This subspecies plays an important role in the ecosystem as an effective seed disperser; its diet is composed mainly of ripe fruits (70–90%), which is key for the regeneration and maintenance of tree diversity in the forests it inhabits (Calle-Rendón *et al*. 2016; Morelos-Juárez *et al*. 2018; Gallo-Viracocha *et al*. 2022). Female spider monkeys have their first offspring between the ages of 7 and 9, with an interbirth interval of 3–4 years, which means that they have a low reproductive rate compared with other primate species (Eisenberg 1973; Milton 1981; Robinson and Janson 1986; Fedigan and Rose 1995).

*A. f. fusciceps* is a priority subject for conservation efforts worldwide, currently listed as one of the world's 25 most endangered primates (Tirira *et al*. 2022) and cataloged as Critically Endangered by the international union for conservation of nature (IUCN) (Moscoso *et al*. 2021). Anthropogenic factors are the main threats to *A. f. fusciceps* populations; as a large mammal with slow growth and reproduction rates, it is affected by the subsistence of hunting practices within indigenous communities, as well as poaching of infants for illegal wildlife trade. However, its most important threat is habitat loss. The Chocó region it inhabits in western Ecuador is a biodiversity hotspot (Myers *et al*. 2000) that requires immediate conservation action, given that it has lost >80% of its original vegetation coverage (Mittermeier *et al.* 2002; Myers *et al*. 2000; Critical Ecosystem Partner Fund, Chocó-Darién-Western Ecuador: Chocó-Manabí Conservation Corridor Briefing Book 2005; Sierra *et al*. 2021). This has led to dramatic population decreases of several species in the region, including the brown-headed spider monkey (Moscoso *et al*. 2021). Furthermore, spider monkeys are highly frugivorous, devoting ∼80% of their time to the consumption of ripe fruits of different tree species. They are, therefore, extremely dependent on low-availability food resources (Di Fiore *et al*. 2008), and this makes them more susceptible to local extinction in areas transformed by humans (Garber *et al*. 2006). The current situation of *A. f. fusciceps* warrants a stronger focus on its conservation to prevent the extinction of the species.

Reductions in the number of individuals in brown-headed spider monkey populations make them susceptible to inbreeding depression and loss of genetic diversity through drift (Frankham 2003; Rivera Román 2017). These 2 processes reduce the species’ resilience to environmental change, thus increasing its vulnerability (Frankham 2003). Whole-genome sequencing (WGS) has been identified as a key tool to manage threatened species, as genomes from representative numbers of individuals can be used to make inferences on a population's demographic history, inbreeding rates, and past genetic bottlenecks, among other significant events (Taylor *et al*. 2022). For a critically endangered species like *A. f. fusciceps*, genomic population studies provide useful information regarding the species’ genetic diversity and population structure, which can assist with the design of adequate management regimes and conservation strategies such as those identified in the Conservation Action Plan for the Ecuadorian Primates (Tirira *et al.* 2018). Population genomic studies require a reference genome, which was not available for *A. f. fusciceps*.

Next-generation sequencing has become more accessible in terms of costs and sequencing velocity. Nevertheless, limited resources in developing countries restrict the accessibility for usage and development of genomic tools (Helmy *et al*. 2016), especially for endangered species in the tropics (regions that harbor at least 50% of the planet's biodiversity; Brancalion *et al*. 2019). Oxford Nanopore sequencing has facilitated genomic research in developing countries with portable, low-cost sequencers that produce ultra-long reads and allow on-site sequencing (Lin *et al*. 2021). While only 1% of all threatened species have a published reference genome (Brandies *et al*. 2019), this could change as access to sequencing technologies increases. Given the overlap of high biodiversity and low accessibility to genomic tools, special emphasis and effort should be placed on genome sequencing projects of endangered species in developing nations.

In the present study, we report the first WGS and assembly of *A. f. fusciceps* using long reads obtained through Oxford Nanopore Technologies.

## Materials and methods

### Sampling

The brown-headed spider monkey individual from which the sample was taken was a subadult male named Mishky, born in the Hacienda Jambelí Rescue Center (2°46′30.48″S 79°44′9.51″O) located in the Guayas province in southwestern Ecuador. In 2014, Proyecto Washu started an ex situ conservation program for the rehabilitation and welfare of this species. The Hacienda Jambelí population of *A. f. fusciceps* is currently considered the largest captive population in Ecuador with a total of 21 individuals: 8 adult males, 1 subadult male, 7 adult females, 1 subadult female, 1 juvenile female, and 3 juvenile males. This population is composed of individuals rescued from the illegal pet trade and others born in the rescue center, as is the case of Mishky.

Mishky was transported to the Tueri Wildlife Hospital (TUERI-USFQ) for medical examination due to injuries sustained while at the Hacienda Jambelí Rescue Center. A 5-ml blood sample was obtained by the TUERI-USFQ veterinarian staff and stored at −80°C in the Laboratorio de Biotecnología Vegetal—USFQ.

### Sequencing methods and preparation

#### DNA extraction

For DNA extraction, the DNeasy Blood and Tissue Kit (QIAGEN, Valencia, CA, USA) was used for 16 total reactions with minor modifications. For the final elution, 30 µl of ultrapure water was used to obtain a total elution of 60 µl after 2 elution steps. The final DNA quantification and quality was assessed with Qubit Fluorometric Quantitation and NanoDrop 2000.

#### Preparation of genomic libraries

The library construction protocol followed the workflow of the Ligation Sequencing Kit SQK-LSK112 (Oxford Nanopore Technologies), which comprises 3 sections. The process started with an average quantity of 2,000 ng per reaction and resulted in a total of 14 libraries. After each section, the DNA concentration was quantified using Qubit Fluorometric Quantitation. The libraries were stored at 4°C awaiting sequencing.

#### Sequencing

Sequencing was carried out in a MinION Mk1C sequencer using 2 R9.4.1 and 6 R10.4.1 flow cells. The 2 R9.4.1 flow cells were used once each for test runs. Each R10.4.1 flow cell was used for 3–4 runs to generate a total of 21 sequencing runs (>24 h). The libraries that had a high DNA quantity (>800 ng) were used for 2 sequencing runs. Similarly, depending on the final concentration of each library, 6, 7, or 12 µl of the sample was loaded to the flow cell, in order to sequence ∼500 ng of DNA. The real-time base calling was executed with Guppy v5.1.13 (ONT), and the resulting output was raw fastq sequencing reads.

### Data processing

#### Initial processing of reads

The raw sequencing reads (.fastq) were first filtered according to quality scores using NanoFilt v2.3.0 (De Coster *et al*. 2018). Reads with quality scores <7 were removed from the analysis (Halstead *et al*. 2021; Feng *et al*. 2022; Petersen *et al*. 2022). Adapters from filtered reads were then trimmed in Porechop v0.2.4 (Wick *et al*. 2017), and sequencing quality was analyzed in Nanoplot v. 1.20.0 (De Coster *et al*. 2018) for both individual sequencing runs and the complete dataset.

#### Assembly, mapping, polishing, and scaffolding

Two different assemblers were used to obtain draft genomes using raw reads. First, SMARTdenovo v.1.0.0 (Liu *et al*. 2021) was used to assemble the obtained reads with the smartdenovo.pl script.

Raw reads were also assembled using Flye v 2.7.1 (Kolmogorov *et al*. 2019), selecting *nano-raw* as the type of input reads and with a specified genome size (g) of 2.6 Gb, based on the reported genome size of the closely related species *A. geoffroyi* (JAKFHY000000000.1) (Shao 2022). The reference genome of *A. geoffroyi* is part of the Whole Genome Shotgun Sequencing Project. It is a contig-level assembly with a 56.87× genome coverage. The sequencing technology used was PacBio RSII, and the reads were assembled with Wtdb2 v.2 (Shao 2022).

Both de novo assembly drafts were mapped against this reference genome using minimap2 v2.24 (Li 2018) to reorder the contigs generated in the assembly. The resulting mapped assemblies were then polished once using Medaka v1.7.2 (Oxford Nanopore Technologies, 2018). The *medaka_consensus* program was employed using the *r103_fast_g507* model.

#### Completeness and quality assessment of genome assembly

Genome assembly quality for both assemblies was evaluated with QUAST v5.2.0 (Mikheenko *et al*. 2018) under default parameters. The reference genome of *A. geoffroyi* (Shao 2022) was specified as the reference for comparison. BUSCO v5.4.4 (Manni *et al*. 2021) was then run using the primates_odb10 database with 13,780 genes to evaluate genome completeness based on expected gene content; we provide statistics for complete, single, fragmented, duplicated, and missing BUSCOs.

#### Genome annotation

The best assembly was selected based on the assembly statistics and BUSCO results, and that assembly was annotated. For genome annotation, a custom repeat library was first created ab initio for the assembled genome of *A. f. fusciceps* using RepeatModeler v2.0.4 (Flynn *et al*. 2020). We applied the “LTRStruct” option for long terminal repeat retroelement identification. Repetitive regions of the genome were identified and soft-masked by RepeatMasker v4.0.7 (Smith *et al.* 2013–2015) in Maker v2.31.9 (Campbell *et al*. 2014). Contigs were then annotated with Maker v2.31.9 (Campbell *et al*. 2014) in 3 consecutive rounds. In the first round, ab initio gene prediction algorithms were run with EST and protein evidence using the *est2genome* and *protein2genome* functions. Reference proteomes from 4 closely related primate species were gathered from the UniProt database (Bateman *et al*. 2021) to be used as protein evidence in Maker (*Sapajus apella*: UP000504640, *Callithrix jacchus*: UP000008225, *Saimiri boliviensis boliviensis*: UP000233220, and *Aotus nancymaae*: UP000233020). EST data were obtained from the NCBI EST database for the most closely related species available (*C. jacchus*). These initial predictions were then used to train the ab initio gene predictor SNAP (Korf 2004), and a second round of Maker was run using the hidden Markov model from SNAP. Finally, a third round of annotation was run with SNAP. Protein and transcript fasta files and gff files generated along the 3 annotation rounds were then merged. To isolate the best-supported gene models, InterProScan v5.61 (Jones *et al*. 2014) was first run to identify conserved Pfam domains on the Maker-predicted proteins. Using accessory scripts from Maker, gene models with annotation edit distance (AED) values >0.5 or lacking Pfam domains were then removed from the gff and fasta files. Finally, the agat_sp_statistics.pl script from the Another Gff Analysis Toolkit software was used to obtain the annotation statistics (Dainat 2020).

#### Foreign contamination screening and elimination

The mapped, polished Flye assembly was screened for foreign contamination using NCBI's FCS-GX tool (Astashyn *et al*. 2023), which identifies contaminant sequences and removes them from the assembled genome. This clean assembly was evaluated using the parameters described in *Completeness and Quality Assessment of Genome Assembly*.

## Results and discussion

### A. *f. fusciceps* assembly

Oxford Nanopore Sequencing of *A. f. fusciceps* produced 55.95 Gb from 8.96 million reads with quality scores greater than q7. Reads greater than or equal to q7 were selected due to the fact that various reports of genome assemblies with Oxford Nanopore reads specify q7 as the threshold for acceptable read quality (Halstead *et al*. 2021; Feng *et al*. 2022; Petersen *et al*. 2022). In order to calculate the coverage, we based our predicted genome size on the closely related species, *A. geoffroyi*, which is 2.6 Gb (Shao 2022). This represents an estimated 21× coverage of the genome. In general, reads had a mean read length of 6.42 kb and a mean read quality score of 10.9 (Table 1).

**Table 1.** Sequencing statistics for the *A. f. fusciceps* genome.

Generated bases	Read count	Coverage	Mean read length	Mean read quality
55.95 Gb	8.96 million	21×	6.42 kb	10.9

The assembly obtained with SMARTdenovo and later polished by Medaka had a total length of 2.58 Gb and contained 6,856 contigs (Table 2). It had an N50 size of 799,988 bp and an L50 of 985, and its largest contig was 5,164,154 bp. When mapped to the reference genome of the closely related *A. geoffroyi*, it had 567.9 mismatches per 100 kb. The Flye assembler alongside the Medaka polisher generated a primary assembly for *A. f. fusciceps* of 2.63 Gb containing 3,861 contigs with an N50 size of 7,560,531 bp (Table 2). The L50 for this assembly was 97, and the largest contig was 44,929,532 bp. In this case, when mapped to *A. geoffroyi*, the assembly had 539.3 mismatches per 100 kb.

**Table 2.** Comparative statistics for the 2 *A. f. fusciceps* draft assemblies generated using SMARTdenovo and Flye, postpolishing with Medaka.

Assembly	Total length	Contig number	N50	Largest contig	L50	# mismatches per 100 kb
SMARTdenovo	2,586,824,631	6,856	799,988	5,164,154	985	567.9
Flye	2,635,867,907	3,861	7,560,531	44,929,532	97	539.3

The Flye assembly is superior to the SMARTdenovo assembly in all analyzed statistics (Table 2). It has a total length similar to the genome size of the closely related *A. geoffroyi* (2.68 Gb; Shao 2022) and less mismatches per 100 kb when compared with this genome. It is much less fragmented, with 3,861 contigs compared with 6,856 in the SMARTdenovo assembly. Furthermore, according to the L50, 50% of the *A. f. fusciceps* genome is represented in 97 contigs in the Flye assembly and in 985 contigs in the SMARTdenovo assembly, proving once again that the SMARTdenovo assembly is less continuous. The Flye assembly also has a much higher N50 and the largest contig size; 50% of the contigs possess a size equal to or longer than 7.56 Mb (Alhakami *et al*. 2017), which is remarkable, since primate species have very large genomes and first assemblies normally produce contig N50 lengths shorter than 100 kb (Jayakumar *et al*. 2021). Finally, the largest contig size of the Flye assembly is 44.9 Mb, almost the size of a human chromosome (Brown 2002).

The assemblers employed in this study possess distinct approaches; SMARTdenovo relies on the Overlap-Layout-Consensus (OLC) algorithm, while Flye uses the generalized de Bruijn Graph (DBG; Wang *et al*. 2021). Primate genomes pose a unique challenge due to their substantial proportion of noncoding regions, rich in repetitive sequences (Ahmad *et al*. 2020). In the context of contig construction where repeats, sequencing errors, and heterozygosity are influential, OLC usually has the advantage because it tolerates these factors by allowing some mismatches in overlap identification. However, DBG excludes these variations on the k-mer graph, making it particularly suitable for large genome assemblies (Li *et al*. 2012). Consistent with our results, Wick and Holt (2021) demonstrated the reliability of the Flye assembler, compared with other assemblers. Their research highlighted its superior performance at low read depths and the minimal occurrence of large-scale sequence errors.

Both genome assemblies were analyzed for annotation completeness based on primate ortholog prediction. The gene database used, primates_odb10, comprises 25 primate genomes and 13,780 genes and is categorized as a high-resolution database, which provides a high level of confidence for genome completeness evaluations (Simão *et al*. 2015; Waterhouse *et al*. 2018). For the SMARTdenovo assembly, we obtained 10,602 (76.9%) complete BUSCOs, of which 10,384 are single copy (75.4%) and 218 (1.6%) are duplicated (1.58%). There were 2,436 (17.7%) missing BUSCOs and 742 (5.4%) fragmented BUSCOs (Supplementary Fig. 1).

When analyzing the Flye assembly, the BUSCO results improved: we obtained more single-copy complete BUSCOs and less missing or fragmented BUSCOs (Supplementary Fig. 1). Specifically, we obtained 11,604 (84.3%) complete BUSCOs, of which 11,362 (82.5%) are single copy and 242 (1.8%) are duplicated (Supplementary Fig. 1). The high number of complete BUSCOs (84.3%) and the low number of duplicated genes indicate a good level of genome completeness and a precise assembly (Simão *et al*. 2015; Manni *et al*. 2021). Regarding the remaining 15.7% of BUSCOs, 564 (4.1%) are fragmented and 1,612 (11.6%) are missing. Technical limitations in gene prediction can inflate the proportions of missing and fragmented BUSCOs, when working with large genomes such as that of *A. f. fusciceps* (Manni *et al*. 2021). Additionally, ONT sequences have error rates of 10–30% that are mainly composed of indels (Morisse *et al*. 2021). However, while the assembly could be improved, the results indicate an overall good quality of the Flye assembly.

**Fig. 1.**
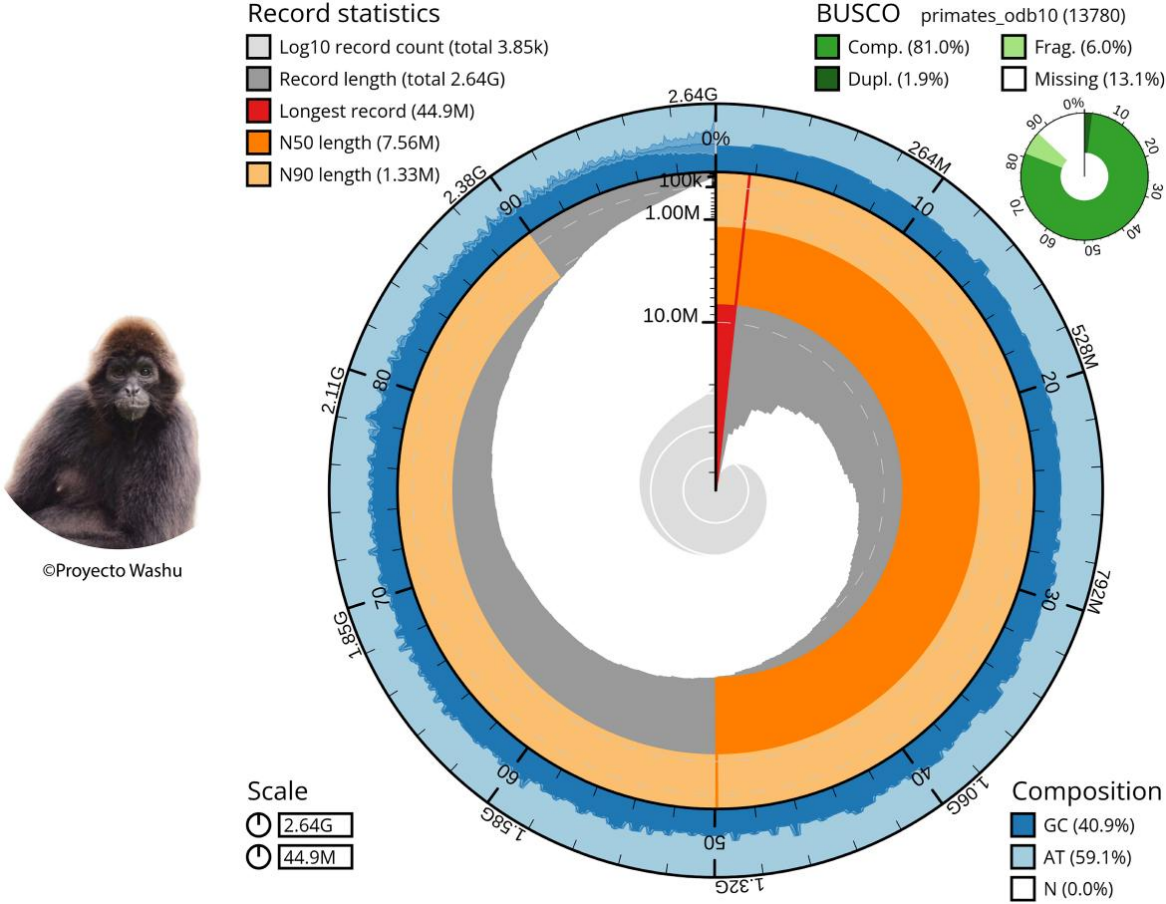
The final genome assembly of *A. f. fusciceps* (Flye assembly) metrics. The BlobToolKit Snailplot shows N50 metrics and BUSCO gene completeness. The plot indicates a total genome size of 2.64 Gb, and a longest obtained contig of 44.9 Mb. The plot also showsthe N50 and N90 values, as well as the GC, AT, and N compositions. A summary of the complete, duplicated, fragmented, and missing BUSCOs (primates_odb10) is represented in the right-hand corner.

Due to the fact that the Flye assembly has better assembly statistics and a more complete annotation, this is the one we selected for further analyses and the one that is reported in this publication. After filtering out foreign contaminations, our *A. f. fusciceps* assembly was compared with that of the closely related *A. geoffroyi* (GCA_023783555.1; Table 3, Fig. 1). This contig-level assembly of *A. geoffroyi* has a total length of 2.68 Gb in 2,732 contigs with a N50 size of 29,212,752 bp and a guanine-cytosine content (GC) content of 40.75%. The values for coverage, contig number, and N50 size for both assemblies were significantly different. However, considering the range of genome size variation among primates (2.09–4.87 Gb; Fantini *et al*. 2016) and that primate genomes’ GC contents are remarkably consistent (Qi *et al*. 2016), the similar values for total length and GC (%) clearly show that this primary genome assembly of *A. f. fusciceps* is adequate, while the differences in coverage, contig number, and N50 suggest there is room for improvement.

**Table 3.** Assembly statistics for the final, clean *A. f. fusciceps* genome assembly compared with the closely related *A. geoffroyi* assembly.

Genome	Total length	Coverage	Contig number	N50	GC (%)
*A. fusciceps*	2,639,265,159	21×	3,851	7,560,531	40.85
*A. geoffroyi*	2,683,028,796	56.87×	2,723	29,212,752	40.75

### Genome annotation

The annotation of the *A. f. fusciceps* assembly in Maker predicted 35,809 protein-coding genes, 88% (31,417) with an AED value <0.5 (Table 4), indicating good protein and transcript evidence support and reasonable quality of the annotation (Sork *et al*. 2016; Saenko *et al*. 2021). AED values closer to 0 generally show greater agreement between the annotation and protein/transcript evidence, while AED values closer to 1 reveal little to no support for the resulting annotation (Eilbeck *et al*. 2009), which is why all gene models with AED values >0.5 were filtered out of the final annotation.

**Table 4.** Summary statistics of the annotated genome (AED < 0.5) of *A. f. fusciceps*.

Statistic	Value
Number of genes	31,417
Number of exons	183,970
Number of introns (in coding sequence [CDS])	149,050
Overlapping genes	715
Mean mRNAs per gene	1.0
Mean exons per mRNA	5.9
Mean introns per mRNA	4.7
Mean gene length (bp)	16,857
Mean exon length (bp)	176
Mean intron length (bp)	3,292
% of genome covered by genes	20.1
% of genome covered by exons	1.2
% of genome covered by introns	18.9

The resulting 31,417 protein-coding genes of *A. f. fusciceps* are comparable with what other mammal genome assemblies have reported like the case of the lowland anoa (*Bubalus depressicornis*) with 32,393 predicted protein-coding genes (Porrelli *et al*. 2022). Nonetheless, gene count is slightly higher than expected when compared with the 22,027 protein-coding genes predicted for *C. jacchus* (GCA_011100555.1; Warren *et al*. 2009) and the 20,350 protein-coding genes for *S. apella* (GCF_009761245.1) (Culibrk *et al*. 2019), both closely related primate species of *A. f. fusciceps*. In general, eukaryotic genomes have around 15,000–25,000 protein-coding genes (Cantarel *et al*. 2008) with the human genome (a primate species) reporting ∼19,100 genes (Piovesan *et al*. 2019). The overestimation of the protein-coding genes could be explained by ONT's long-read accuracy limitations compared with other sequencing technologies (Rang *et al*. 2018), though the resulting annotation of our genome still shows an accurate prediction. Additionally, since only soft masking was used for repeat masking during MAKER annotation, it is possible that repetitive regions were misconceived as putative genes (Saenko *et al*. 2021), increasing the predicted number of coding sequences.

Furthermore, the annotation of the *A. f. fusciceps* genome predicted a mean gene length of 16,857 bp (Table 4), a length comparably smaller to what has been reported for other closely related primate species, with mean gene lengths of ∼40,000 bp (Warren *et al*. 2009; Culibrk *et al*. 2019; Harris *et al*. 2020). The same pattern is evident when we compare mean intron length (3,292 bp) and mean exon length (176 bp). These differences can likely be attributed to the level of fragmentation of our genome and the inaccurate prediction of genomic features in repetitive regions. This is expected since around 50% of a primate genome is covered by repetitive elements (Rogers and Gibbs 2014), making the annotation of other genomic features a challenging task (Okazaki and Hume 2003). Nonetheless, differences in genomic feature predictions between closely related species have been reported in other reference genomes (Jiang *et al*. 2022; Kaur *et al*. 2023) and could be attributed to the sequencing technology used and the level of genome fragmentation.

### Importance of reference genome

Numerous studies have established the importance of genomic data to understand the evolutionary history of a species and to develop appropriate conservation and management strategies (Kleinman-Ruiz *et al*. 2017; Saremi *et al*. 2019; Kenny *et al*. 2020; Nong *et al*. 2021; Pfenninger *et al*. 2021). WGS leads to a better understanding of the biology of a species and provides insights into fundamental processes that shape their evolution (Ryder 2005), and its application can provide important and accurate information about its demographic history, admixture, introgression, recombination, linkage disequilibrium, genomic regions evolving under selective pressures, and other evolutionary processes (Theissinger *et al*. 2023). For critically endangered species like the brown-headed spider monkey, genomic approaches are even more valuable due to the scarcity of samples for genetic studies; therefore, WGS maximizes the information that researchers can harness from each sample. However, in order to be able to generate and fully take advantage of this information, a reference genome is required (Theissinger *et al*. 2023).

Species under such conservation threats face a dire need for conservation actions to reverse their declining population trends. Currently, Proyecto Washu is deepening the understanding of the brown-headed spider monkey's behavior and ecology through observational studies of a population of spider monkeys living in a highly fragmented landscape. The sequencing of its genome provides an opportunity to improve its conservation through the development of population-level studies to evaluate its genetic diversity and gene flow. Moreover, genetic population studies may allow us to better differentiate its populations, perform identification of individuals and kinship patterns, evaluate the dispersion and migration of individuals, and identify and prioritize biological corridors through which monkey populations move. Biological corridors prevent the isolation of populations in closed forest fragments, which reduces inbreeding and helps to maintain genetic diversity in the area (Kirchner *et al*. 2003; Haddad *et al*. 2015).

While major progress has been made in animal genome sequencing in the last 25 years, significant gaps and biases remain in geographic and taxonomic representation resulting in an improper depiction of the global genetic pool (Hotaling *et al*. 2021). Ecuador, for instance, has a limited record of genetic and genomic research (Zambrano-Mila *et al*. 2019) despite its sizable biodiversity (Celi and Villamarín 2020). This is a multifaceted issue resulting from the lack of sequencing platforms and training in genome data analysis and research costs (Hotaling *et al*. 2021). This makes outsourcing a popular alternative to generate genomic sequences, despite the limitations of using third-party service providers (Helmy *et al*. 2016). A feasible pathway to democratize sequencing efforts and to involve developing countries is through the usage of portable sequencing devices such as the Oxford Nanopore Technologies MinION, as applied in this study. This is a time-efficient and cost-efficient technology for the assembly of all genome sizes (Wang *et al*. 2021), which operates on standard computing resources. Its long-read length and portability enable the use of these devices in basic research (e.g. assembly of preliminary nonmodel organism genomes), clinical usage, and on-site applications (Wang *et al*. 2021). Due to its ease of use and convenience, the current report represents an initial sequencing project, which will be further extended to other underrepresented Ecuadorian mammals. We expect that this and similar efforts will generate critical information for future genomic studies directed toward conservation and management efforts.

### Conclusion

The brown-headed spider monkey (*A. f. fusciceps*) is a critically endangered primate species, facing multiple threats such as habitat loss and hunting, emphasizing the urgent need for conservation efforts. WGS has been identified as a crucial tool for managing threatened species. Here, we present the first WGS and assembly of *A. f. fusciceps* using long reads obtained through Oxford Nanopore Technologies, which resulted in a good-quality assembly. The genomic insights gained from this study provide valuable information, which can lead to the development of tools for the conservation of *A. f. fusciceps*. Moreover, the pipelines used in this study can serve as a foundation for sequencing and assembling genomes of other endangered species in developing nations, ultimately aiding in the preservation of global biodiversity.

## Supplementary Material

jkae014_Supplementary_Data

## Data Availability

The raw reads, genome assembly, and annotation can be found at GSA figshare: https://doi.org/10.25387/g3.24076638. This Whole Genome Shotgun project has been deposited at DDBJ/ENA/GenBank under the accession JAZHEH000000000. The version described in this paper is version JAZHEH010000000. ONT long-read raw sequences have been deposited in the NCBI Sequence Read Archive database under BioProject PRJNA1009451. The script used for assembly and annotation is described in protocol.io at the following https://doi.org/10.17504/protocols.io.6qpvr3892vmk/v1. Supplemental material available at G3 online.
