# Supplementary material for: First whole-genome sequence and assembly of the Ecuadorian brown-headed spider monkey (Ateles fusciceps fusciceps), a critically endangered species, using Oxford Nanopore Technologies
Source: G3 (Bethesda). 2024 Jan 19;14(3):jkae014. doi: 10.1093/g3journal/jkae014 (PMC10917520; doi:10.1093/g3journal/jkae014)
Supplement: jkae014_Supplementary_Data [file jkae014_supplementary_data.docx]

**Supplementary Data**


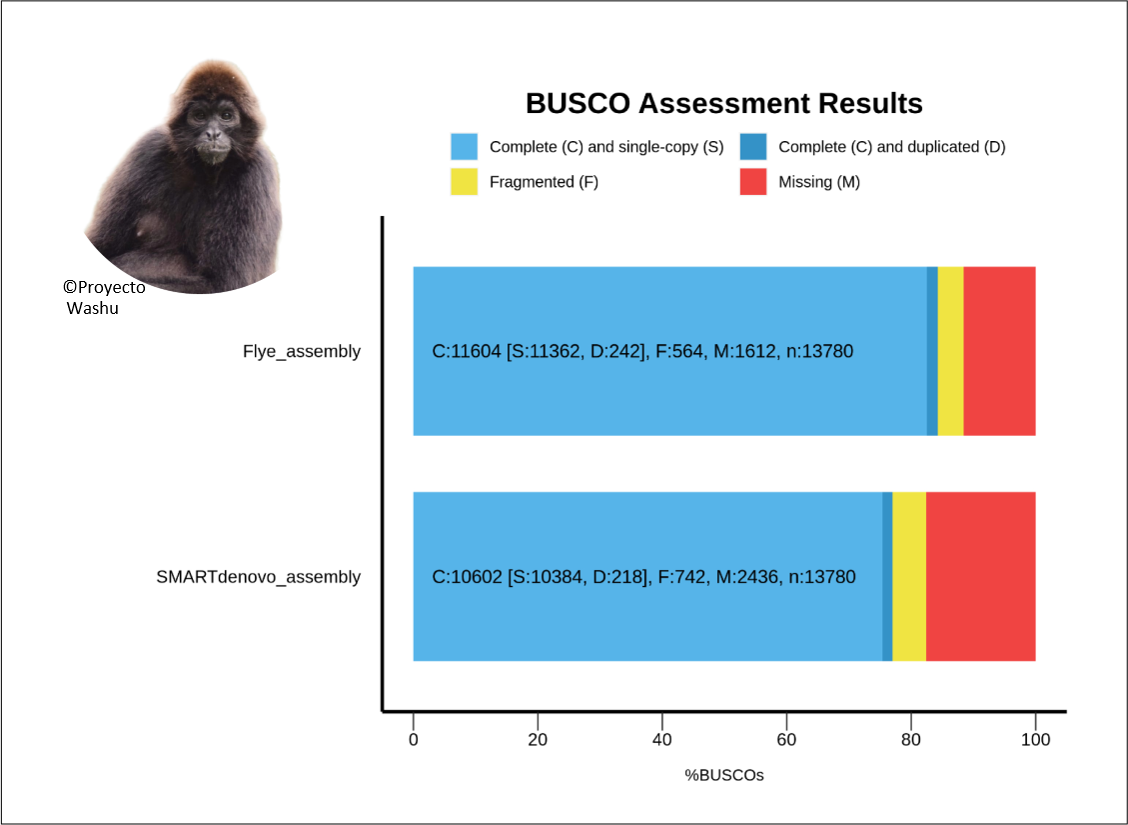


**Figure S1**. BUSCO results for SMARTdenovo and Flye *A.f.fusciceps* assemblies. Complete and Single-Copy, Complete and Duplicated, Fragmented and Missing BUSCOS are presented. Primates_odb10, containing 13,780 genes, was used as the reference database.
